# Trace Element Speciation in Food: A Combined Enzymolysis—SEC-ICP-MS Approach

**DOI:** 10.6028/jres.093.072

**Published:** 1988-06-01

**Authors:** H. M. Crews, R. Massey, D. J. McWeeny, J. R. Dean

**Affiliations:** Ministry of Agriculture, Fisheries and Food, Norwich, U.K.; The Polytechnic, Plymouth, U.K.

Trace elements in food can be either desirable, tolerable or undesirable. They occur in the diet as natural constituents of agricultural produce, as deliberate additives and as contaminants from environmental and processing sources. Historically, their significance to man has been assessed on the basis of total concentration of the element in the food item—or the total amount in the diet as a whole (with the exception of the measurement of mercury and to a lesser extent arsenic where specific forms are sometimes measured). In recent years the inadequacy of simply measuring the total concentration has been increasingly appreciated and efforts to provide information which is targeted more accurately to the needs of the nutritionist and the toxicologist are now being made.

Over the last few years, the MAFF Food Science Laboratory has had a small programme investigating ways of obtaining more meaningful information about the chemical forms of trace elements in food. As a first stage of a general approach to the problem, attention has been restricted to species which are soluble at around neutral pH after treatment with normal monogastric mammalian digestive enzymes. A procedure has been developed in which food is treated sequentially with pepsin at around pH 2 and with pancreatic enzymes at around pH 7 at 37 °C. A limitation of this procedure is that it is a “static” system, whereas absorption is dynamic; if there is equilibrium between soluble and insoluble species, the system will underestimate the amount which is potentially available biologically.

In some very simple experiments, food composites representing the cereal, meat, fish and green vegetable components of the UK diet were examined with and without the addition of small amounts of copper, zinc, cadmium and lead as inorganic salts. The properties of the endogenous element which was soluble after enzyme treatment has been compared with that of the added element. For all the foods studied, endogenous and added copper behaved similarly, but there were marked differences for some of the other elements. For instance, zinc from fish is largely insoluble, and added zinc also becomes insoluble; other foods release much zinc in soluble form. On the other hand, iron from meat is largely soluble but added iron becomes insoluble [1].

This investigation has been developed further in studies of the effect of one food upon the release of trace elements from another. The amount of acid-soluble cadmium available from crab meat is reduced by 75% by digesting it along with wholemeal bread; white bread has very little effect. Similar experiments show that addition of 10% soya to ground beef gives a much lower release of zinc from the beef—presumably a soya phytate effect. Processing of beef can affect the solubility of iron; inorganic iron as taken in dietary supplements is much more soluble if digested along with cereals and vegetables than with meat and fish [2].

The broad conclusions of these experiments were that: a) acidity enhances solubility, b) enzyme action changes solubility, c) processing can affect solubility, and d) presence of other foods can affect solubility. Even where an element was soluble, there were indications of changes in chemical form as a result of enzyme action, processing or the presence of other foods.

In devising an investigation of these changes, the criteria were that it should be, i) with foods containing levels of toxic elements typical of those in the normal food supply; ii) with systems which, so far as possible, did not alter the chemical form of the element naturally present. The first point created problems, i.e., natural levels are close to the limits of reliable determination by atomic absorption spectrometry; dilution occurring on enzymolysis and separation of species produced nonmeasurable levels. One solution to this problem lies in the high sensitivity of ICP-MS [3] and the ability to use ICP-MS as a detector with LC columns.

Particular attention has been paid to steric exclusion chromatography as one of the most gentle forms of separation achievable. It is also a relevant separation of soluble macro molecules (which are unlikely to be absorbed) from lower molecular weight species which are more likely candidates for absorption. Additionally, some work with reversed-phase HPLC linked to ICP-MS has also been carried out.

With HPLC systems a guard column is used to protect the analytical column; post-column injections of standards are used for calibration checks. To minimize peak-broadening, aerosol transfer distance should be reduced by mounting the nebulizer system close to the torch. Flow rates above 0.5 to 0.7 mL/min can lead to signal instability unless precautions against condensation are taken. In an early piece of work, the cadmium content of crab meat with separation by HPLC and monitoring by UV and ICP-MS showed that the major protein peak has almost completely eluted before the cadmium peak corresponding to a somewhat lower molecular weight cadmium-containing protein.

More recent studies of the HPLC separation of the proteins from pig kidney before and after cooking show that in the raw state there are three peaks—the higher molecular weight materials disappear during cooking, i.e., are rendered insoluble by heat denaturation, whereas the heat-resistant metallothionein remains intact.

Another recent study concerns an organo-arsenical drug used in poultry production. In this, reversed-phase HPLC has been linked to the ICP-MS in much the same way for HPLC. This system allows good separation of “Roxarsone” from other low molecular weight substances and from interference from molecular ions formed from argon in the plasma in the presence of chloride ion.

Arsenic in extracts from muscle of chickens fed on a base diet and one supplemented with “Roxarsone” have been compared. The “Roxarsone” peaks correspond to arsenic concentrations of 7 and 10 ng/g in the muscles from treated birds and demonstrates that in the untreated birds the concentration is less than 2 ng/g.

The sensitivity foreseen with these systems is now a matter of reality rather than potential—and the ability to use it in speciation studies at tissue concentrations at the ng/g level is a matter of fact rather than of speculation.
